# Impact of Physical Activity on Stroke Risk in Middle‐Aged and Older Adults

**DOI:** 10.1002/brb3.71601

**Published:** 2026-07-14

**Authors:** Huiping Bai, Zetai Bai, Guanzhao Wu

**Affiliations:** ^1^ Qilu Hospital (Qingdao), Cheeloo College of Medicine Shandong University Qingdao China; ^2^ Qilu Hospital, Cheeloo College of Medicine Shandong University Jinan China; ^3^ Department of Central Laboratory, Qilu Hospital (Qingdao), Cheeloo College of Medicine Shandong University Qingdao China

**Keywords:** cardiovascular biomarkers, CHARLS, physical activity, stroke

## Abstract

**Purpose:**

The relationship between physical activity intensity and the risk of first‐ever stroke in middle‐aged and older adults remains incompletely understood. This study aimed to investigate the association between different intensities and total volumes of physical activity and the incidence of first stroke, and to explore potential cardiovascular mediators underlying this relationship.

**Method:**

This prospective cohort study utilized data from the China Health and Retirement Longitudinal Study (CHARLS), including 4380 stroke‐free adults aged ≥ 45 years at baseline. Physical activity was assessed using the International Physical Activity Questionnaire Short Form and classified into sedentary behavior, active physical activity, and highly active physical activity. Total physical activity was quantified in metabolic equivalent task minutes per week (MET‐min/week). Incident stroke was identified through self‐reported physician diagnosis during a 7‐year follow‐up period. Cox proportional hazards models, multivariable logistic regression, restricted cubic spline analyses, subgroup analyses, and mediation analyses were conducted after adjusting for demographic, lifestyle, and clinical confounders.

**Finding:**

During follow‐up, 300 participants (6.8%) experienced a first‐ever stroke. Highly active physical activity was associated with a significantly reduced risk of first stroke compared with sedentary behavior (adjusted hazard ratio [HR], 0.52; 95% confidence interval [CI], 0.30–0.92; adjusted odds ratio [OR], 0.45; 95% CI, 0.36–0.55). A nonlinear, flipped *J*‐shaped dose–response relationship was observed between total physical activity and stroke risk, with the lowest risk occurring at approximately 13,045 MET‐min/week. Subgroup analyses showed stronger protective effects of highly active physical activity in men, individuals without heart disease, overweight individuals, and those with hypertension. Mediation analyses indicated that the protective association was largely mediated by improvements in cardiovascular indicators, including blood pressure, high‐density lipoprotein cholesterol, body mass index, and the triglyceride–glucose index.

**Conclusion:**

The inverse association between highly active physical activity and first stroke was partially accounted for by cardiovascular biomarkers.

## Graphical abstract

Highly active physical activity significantly reduces the risk of first‐ever stroke in middle‐aged and older adults, with cardiovascular improvements‐including lower blood pressure, healthier lipid profiles, and better metabolic status‐partially mediating this protective effect.

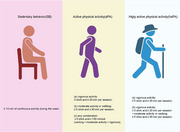

## 1 Introduction

Stroke, as one of the leading causes of long‐term disability and death in both developing and developed countries, has its incidence significantly influenced by lifestyle factors, including intense physical exercise (Tu et al. 2023; Ebinger et al. 2021; Y.Wang et al. 2021). Although numerous studies have highlighted the protective role of exercise against cardiovascular diseases (CVDs), the specific relationship between the intensity of physical activity and the risk of first stroke in middle‐aged and older adults remains unclear (Ramakrishnan et al. 2021; Garcia et al. 2023; Mehta et al. 2020).

Previous research has indicated that engaging in moderate to high levels of physical activity is more effective in preventing stroke compared to lower levels of activity in men (Kiely et al. 1994). In addition, a retrospective cross‐sectional study investigated factors associated with stroke severity and found that physical activity levels prior to the stroke were inversely related to both stroke severity and short‐term outcomes (Christensen et al. 2005). Physical activity is one of the most important actions people of all ages can engage in to improve their health (Piercy et al. 2018). Physical activity has been associated with improved endothelial function, attributed in part to increased nitric oxide (NO) bioavailability (De Nigris et al. 2003; Kauser et al. 2000, Kingwell 2000; Leaf et al. 1999; Leeuwenburgh and Heinecke 2001). A cohort study of Chinese adults without prior CVDs found that higher occupational or nonoccupational physical activity is significantly associated with a reduced risk of severe CVDs (Bennett et al. 2017). In addition, a cohort study from 21 high‐income, middle‐income, and low‐income countries found that physical activity is second only to tobacco use among behavioral risk factors related to CVDs (Yusuf et al. 2020). Despite the emphasis on exercise's protective effects, the specific relationship between physical activity intensity and the risk of first stroke in middle‐aged and older adults is still not well understood (Ramakrishnan et al. 2021; Mehta et al. 2020; Garcia et al. 2023).

In this cohort study of middle‐aged and older individuals, we explore the association between physical activity intensity and the risk of first stroke. Furthermore, we identified the total amount of physical activity associated with the lowest risk of first stroke. Through longitudinal and cross‐sectional studies, we found that highly active physical exercise is associated with a reduced risk of first stroke, mediated by cardiovascular biomarkers, further investigating the direct and indirect relationships between highly active physical exercise and the risk of first stroke. These findings are expected to provide recommendations for physical activity in middle‐aged and older individuals, reducing the risk of initial stroke.

## 2 Methods

### 2.1 Study Population

The China Health and Retirement Longitudinal Study (CHARLS) aims to establish a public, high‐quality, nationally representative micro‐database. All data collected by CHARLS are made available to researchers worldwide via the official CHARLS website at https://charls.pku.edu.cn/. This study primarily relies on the CHARLS health status and function questionnaire, encompassing self‐rated health and physical examination indicators. Baseline blood sample collection was conducted during the CHARLS from 2011 to 2012. For blood samples, the initial tube is allocated for complete blood count (CBC) testing, while the remaining two tubes are utilized for plasma extraction and hemoglobin A1C (HbA1c) testing. These samples are then transported to the Chinese Center for Disease Control and Prevention in Beijing within a 2‐week period. Ethical approval for the study was obtained from the Biomedical Ethics Review Committee of Peking University (IRB00001052‐11015), and all participants provided informed consent prior to inclusion. Informed consent was obtained from all subjects and/or their legal guardian(s). All methods were carried out in accordance with relevant guidelines and regulations.

### 2.2 Outcome Ascertainment

Participants who were non‐stroke at baseline and reported a stroke during subsequent follow‐up were recorded as incident cases. Data on the occurrence of stroke were systematically collected through a questionnaire, asking whether the participants had been diagnosed with a stroke by a physician, the date of diagnosis or their awareness of the condition, and whether they were receiving treatment for stroke. The date of the first stroke occurrence was recorded as the midpoint between the date of the interview reporting the first stroke event and the date of the previous interview.

### 2.3 Exposure Assessment

**FIGURE 1:**
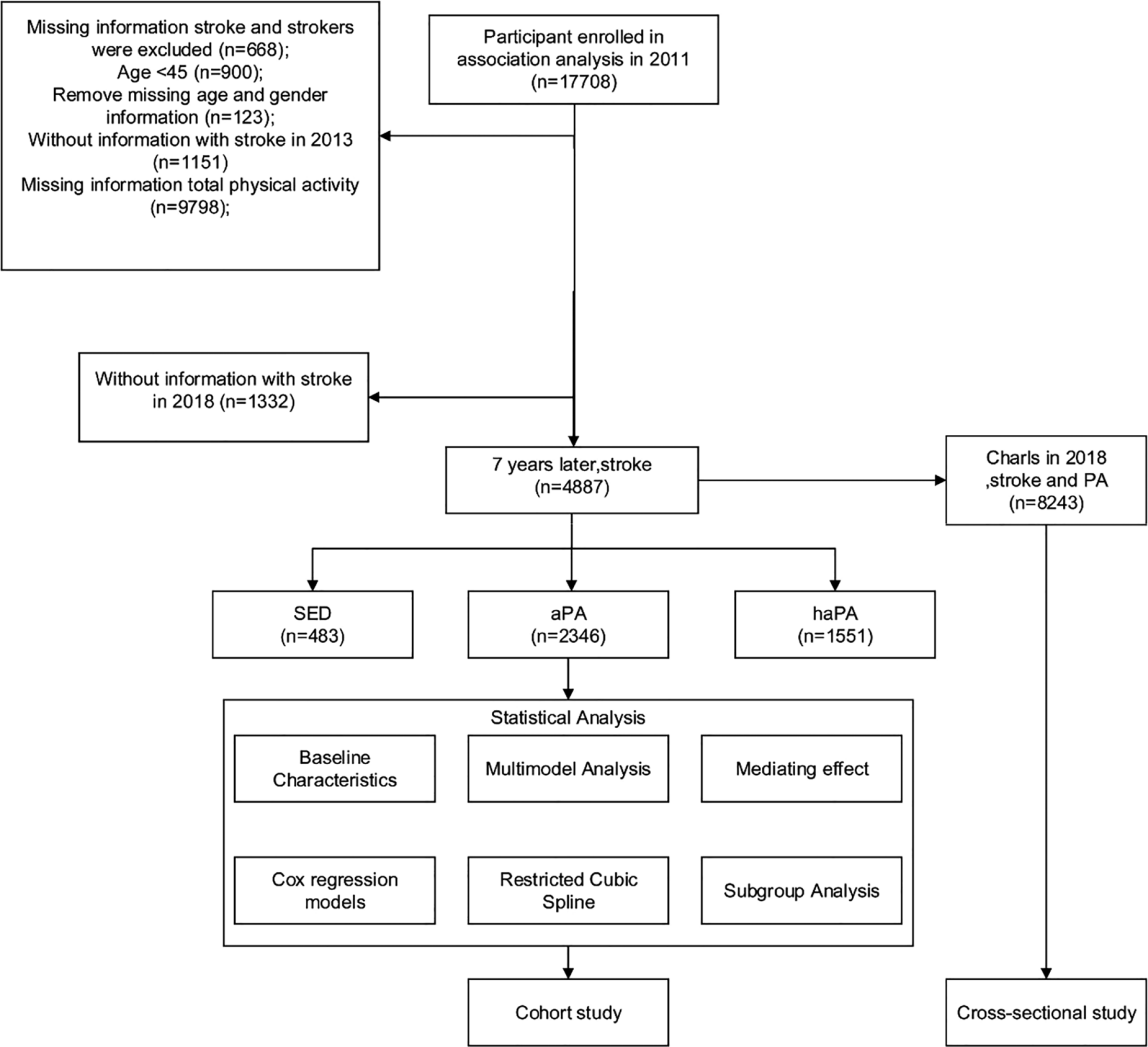
The flow chart of the study. aPA, active physical activity; haPA, highly active physical activity; SB, sedentary behavior.

This study utilized the International Physical Activity Questionnaire Short Form (IPAQ‐SF) to guide the standardized collection of physical activity data. Physical activity was categorized into three intensity levels: Vigorous physical activity, such as plowing and aerobics, which typically results in shortness of breath; moderate physical activity, such as mopping the floor or practicing tai chi, which leads to breathing faster than usual; and light physical activity, which includes daily walking at work or at home and walking for recreational, sport, exercise, or entertainment purposes. Due to the lack of specific durations for each activity type, the median of each physical activity time range was used for conversions, while durations greater than 4 h were counted as 240 min. Total physical activity was measured by summing all types of activity, expressed in metabolic equivalent minutes per week (MET‐min/week), with the formula: Total physical activity = 8.0 × vigorous physical activity duration per week + 4.0 × moderate physical activity duration per week + 3.3 × light physical activity duration per week. Physical activity was assessed at baseline (Wave 1, 2011). In addition, subjects were divided into three groups based on the intensity, frequency, and duration of physical activity, with the grouping criteria detailed in Figure 1.

### 2.4 Covariates

Covariates were selected based on prior research and clinical expertise, and include the following: Age, gender, marital status, residence, smoking status, alcohol consumption, educational level, chronic kidney disease, heart disease, dyslipidemia, diabetes, serum high‐density lipoprotein cholesterol (HDL‐c), body mass index (BMI), total serum cholesterol (TC), serum triglycerides (TG), fasting plasma glucose (FPG), systolic blood pressure (SBP), and estimated glomerular filtration rate (eGFR). The eGFR was calculated using the updated 2021 CKD‐EPI equation, which is designed to reduce racial bias.

### 2.5 Statistical Analysis

For baseline characteristics, we compared categorical variables between SB, aPA, and haPA using chi‐square tests, and continuous variables using one‐way ANOVA or Wilcoxon tests. Continuous variables are presented as means ± standard deviation (SD), and categorical variables are presented as percentages. We used Cox proportional hazards regression models to derive hazard ratios (HRs) and 95% confidence intervals (CIs), with sedentary behavior as the reference group. Confounding factors such as age, sex, marital status, alcohol consumption, smoking status, education level, and history of diabetes and cancer were adjusted to study the relationship between different types of physical activity and the risk of first stroke. Multiple imputation was used for missing values. To explore the potential nonlinear relationship between total physical activity and the risk of first stroke, we analyzed the relationship in different populations using restricted cubic spline functions. For mediation analysis, causal stepwise regression testing and the product of coefficients method were used to analyze the indirect effects of different cardiovascular indicators between highly active physical activity (haPA) and the first stroke. Interaction effects between haPA and stratification variables were tested using a likelihood ratio test with multiplicative interaction terms. Statistical analyses were performed using SPSS version 25.0 for Windows; data cleaning and graphing were conducted using Stata 15.1 (StataCorp) and R version 3.5.2 (R Foundation for Statistical Computing). All significance tests were two‐sided, and the results were considered statistically significant at *p* < 0.05.

### 2.6 Ethics Approval and Consent to Participate

The study was approved by the ethics review committee (institutional review board) of Peking University. Informed consent was obtained from all subjects and/or their legal guardian(s). All methods were performed in accordance with relevant guidelines and regulations. All data are openly published as microdata at http://opendata.pku.edu.cn/dataverse/CHARLS with no direct contact with all participants.

## 3 Results

**FIGURE 2:**
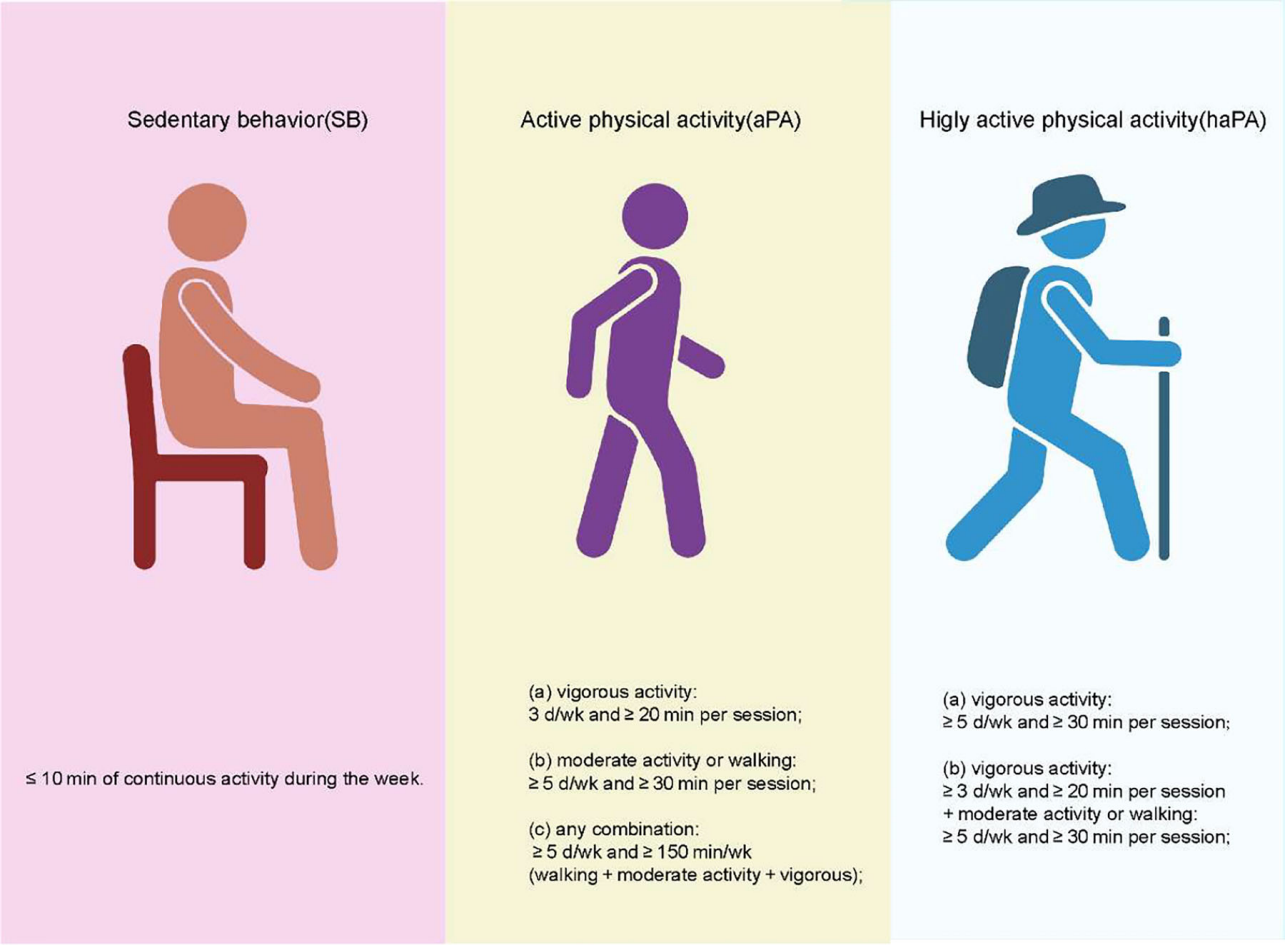
Criteria for classifying physical activity.

Between 2011 and 2018, a total of 4380 middle‐aged and older adults were enrolled in the study. Exclusions comprised 3151 individuals lacking stroke information or with a history of stroke, 900 individuals aged below 45, 123 individuals without data on age and gender, and 9798 individuals lacking information on total physical activity levels (Figure 2 ). Significant data omissions included blood test indicators, notably TC, which was absent for 27% of participants.

Table 1 presents demographic data and laboratory markers for groups stratified by sedentary behavior (SB, *n* = 483), active physical activity (aPA, *n* = 2346), and haPA (*n* = 1551). Relative to the SB group, participants in the haPA group exhibited lower rates of heart disease, diabetes, and dyslipidemia. Demographically, compared to the SB group, the haPA group comprised a higher proportion of females, a younger age profile, and a higher prevalence of rural residency. Other baseline characteristics, including smoking, alcohol consumption, and laboratory indices, also displayed variations (Table 1).

**TABLE 1:** Baseline characteristics of study participants in the CHARLS, 2011–2018: A study on physical activity and stroke.

	No. (%)					
Characteristic	Total (*n* = 4380)	SB (*n* = 483)	aPA (*n* = 2346)	haPA (*n* = 1551)	Statistic	*p*
Age, mean ± SD	58.40 ± 8.49	60.11 ± 9.56	59.08 ± 8.75	56.83 ± 7.43	*F* = 44.86	**< 0.001**
Sex					*χ* ^2^ = 98.66	**< 0.001**
Male	2383 (54.41)	299 (61.90)	1396 (59.51)	688 (44.36)		
Female	1997 (45.59)	184 (38.10)	950 (40.49)	863 (55.64)		
Marry					*χ* ^2^ = 16.69	**< 0.001**
Other	437 (9.98)	55 (11.39)	266 (11.34)	116 (7.48)		
Married	3943 (90.02)	428 (88.61)	2080 (88.66)	1435 (92.52)		
Area					*χ* ^2^ = 155.70	**< 0.001**
Urban	1564 (35.71)	190 (39.34)	1008 (42.97)	366 (23.60)		
Rural	2816 (64.29)	293 (60.66)	1338 (57.03)	1185 (76.40)		
History						
Diabetes	229 (5.28)	32 (6.64)	146 (6.29)	51 (3.32)	*χ* ^2^ = 18.34	**< 0.001**
Heart disease	483 (11.07)	58 (12.06)	332 (14.19)	93 (6.03)	*χ* ^2^ = 63.35	**< 0.001**
Dyslipidemia	416 (9.67)	47 (9.81)	273 (11.86)	96 (6.31)	*χ* ^2^ = 32.33	**< 0.001**
Kidney disease	233 (5.34)	19 (3.96)	127 (5.43)	87 (5.65)	*χ* ^2^ = 2.14	0.343
Alcohol consumption					*χ* ^2^ = 50.74	**< 0.001**
None	2694 (61.55)	330 (68.46)	1517 (64.69)	847 (54.65)		
Former	1683 (38.45)	152 (31.54)	828 (35.31)	703 (45.35)		
Smoking status					*χ* ^2^ = 70.47	**< 0.001**
None	2757 (62.95)	328 (67.91)	1581 (67.39)	848 (54.67)		
Former	1623 (37.05)	155 (32.09)	765 (32.61)	703 (45.33)		
Education level					*χ* ^2^ = 43.37	**< 0.001**
Pre‐elementary	2038 (46.57)	243 (50.31)	1035 (44.16)	760 (49.06)		
Elementary school	924 (21.12)	85 (17.60)	491 (20.95)	348 (22.47)		
Middle school	914 (20.89)	108 (22.36)	488 (20.82)	318 (20.53)		
High school and beyond	500 (11.43)	47 (9.73)	330 (14.08)	123 (7.94)		
TC, mean ± SD	193.09 ± 38.13	196.59 ± 40.88	193.95 ± 37.22	190.78 ± 38.47	*F* = 4.06	**0.017**
TG, mean ± SD	133.52 ± 106.90	143.91 ± 112.22	138.57 ± 98.72	123.02 ± 115.53	*F* = 9.22	**< 0.001**
HDL, mean ± SD	51.13 ± 15.45	48.49 ± 14.80	49.53 ± 14.58	54.26 ± 16.35	*F* = 39.05	**< 0.001**
eGFR, mean ± SD	96.65 ± 13.06	95.39 ± 14.34	95.92 ± 13.05	98.09 ± 12.56	*F* = 11.36	**< 0.001**
SBP, mean ± SD	128.60 ± 20.76	133.18 ± 23.54	128.78 ± 20.62	126.98 ± 19.86	*F* = 14.25	**< 0.001**
BMI, mean ± SD	24.09 ± 28.32	23.96 ± 4.08	24.91 ± 38.63	22.94 ± 3.58	*F* = 1.96	0.141

Abbreviations: aPA, active physical activity; BMI, body mass index; eGFR, estimated glomerular filtration rate; haPA, highly active physical activity; HDL, high‐density lipoprotein; SB, sedentary behavior; SBP, systolic blood pressure; TC, total cholesterol; TG, triglyceride.

**FIGURE 3:**
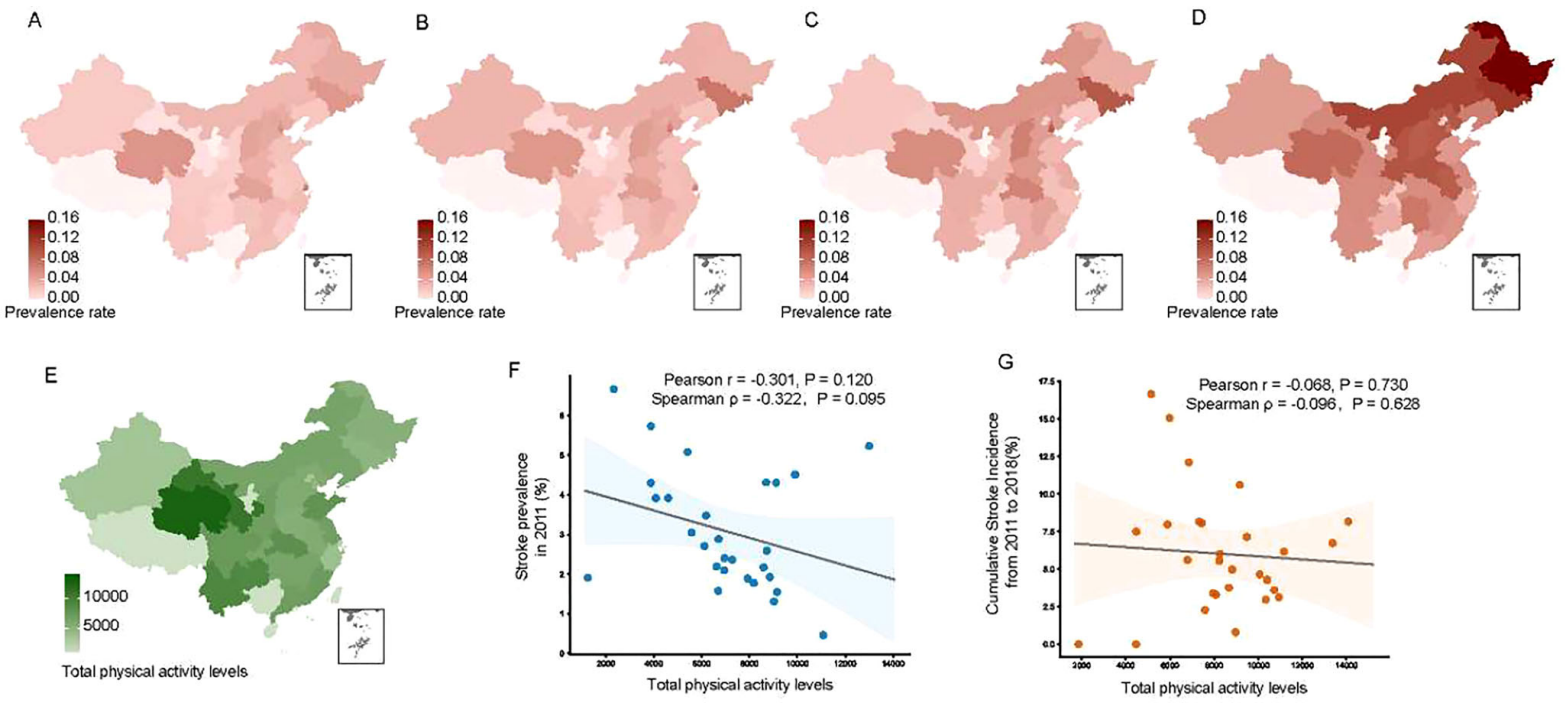
To study the regional distribution of stroke prevalence in the map of China. (A). Stroke prevalence in 2011 (B). Stroke prevalence in 2013 (C). Stroke prevalence in 2015 (D). Stroke prevalence in 2018. (E) Physical activity levels in 2011. (F) Provincial‐level ecological correlation between physical activity and stroke prevalence in 2011 (Pearson *r* = −0.301, *p* = 0.120; Spearman *ρ* = −0.322, *p* = 0.095). (G) Provincial‐level ecological correlation between physical activity in 2011 and cumulative stroke incidence (2011–2018) (Pearson *r* = −0.068, *p* = 0.730; Spearman *ρ* = −0.096, *p* = 0.628). Solid lines: linear fits. Shaded areas: 95% confidence bands.

Figure 3 illustrates a regional map depicting the prevalence of stroke across various regions of China. The findings from this study reveal marked regional disparities in stroke prevalence rates (PR) between northern and southern areas. Specifically, the PR is significantly higher in northern regions compared to the south. However, despite the overall higher PR in the north, economically developed areas such as Beijing and Tianjin exhibit lower incidence rates than other northern regions. In general, the PR in southern China remains consistently lower than in the north. The two regions with the most substantial increases in PR were Heilongjiang Province, where the rate rose from 0.039 in 2011 to 0.159 in 2018, and the Inner Mongolia Autonomous Region, where it increased from 0.030 to 0.110 during the same period. In contrast, regions with the smallest changes in PR were Shanghai, which saw a decline from 0.067 to 0.035, and Beijing, where the rate decreased from 0.043 to 0.019.

Provincial‐level mean physical activity in 2011 showed no statistically significant ecological correlation with either stroke prevalence in 2011 (Pearson *r* = −0.301, *p* = 0.120) or 7‐year cumulative stroke incidence from 2011 to 2018 (Pearson *r* = −0.068, *p* = 0.730).

**FIGURE 4:**
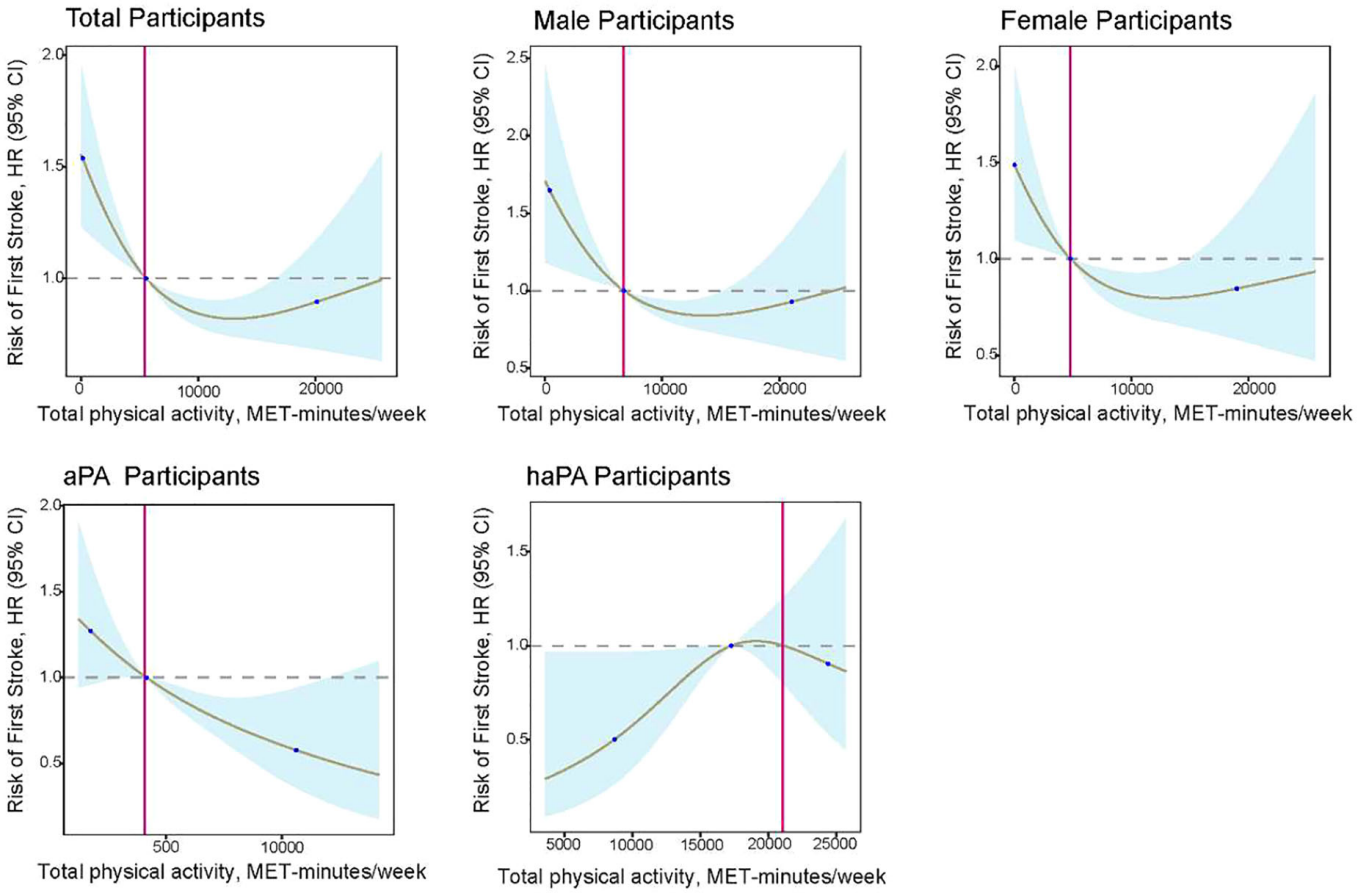
Dose–response relationship between total physical activity and stroke. The solid line represents the hazard ratio, and the shaded area represents the 95% confidence interval (CI). A restricted cubic spline regression model was set with three knots at the 10th, 50th, and 90th percentiles of the total physical activity.

The restricted cubic spline analysis demonstrated a linear dose‐response relationship between aPA and the risk of a first stroke. Specifically, among individuals engaging in aPA, the risk of a first stroke decreased as the intensity of aPA increased, indicating an inverse correlation between aPA intensity and stroke risk. In the RCS regression, there was a significant nonlinear relationship between total physical activity and the risk of a first stroke (*p* = 0.007, Figure 4 and Figure S1), displaying a flipped J‐shaped dose‐response curve. Beyond the inflection point of 13,045 MET‐min/week, the HR for a first stroke increased with greater total physical activity. Simultaneously, a flipped J‐shaped nonlinear relationship was also observed between total physical activity and the risk of a first stroke in men.

Cross‐sectional studies demonstrated the association between types of physical activity and the risk of the first stroke in 2018, with adjustments for confounding factors. Multivariable logistic regression models demonstrated that both aPA (OR: 0.65, 95% CI, 0.56–0.76) and haPA (OR: 0.45, 95% CI, 0.36–0.55) were associated with reduced first stroke risk (Table S1). Conversely, a cross‐sectional study conducted in 2015 indicated a reduced risk of first stroke solely with aPA (OR: 0.70, 95% CI, 0.50–0.98) (Table S2).

Table 2 illustrates the HRs with corresponding 95% CIs for the relationship between different types of physical activity and the risk of initial stroke. Participants engaged in haPA exhibited a diminished risk of stroke, with an HR of 0.6 (95% CI, 0.41–0.86). Following adjustment for confounding factors in Model 3, the risk of the first stroke was 0.67 (95% CI, 0.46–0.99).

**TABLE 2:** The relationship between types of physical activity and the risk of first stroke in the general population.

Variables	Model 1		Model 2		Model 3	
	HR (95% CI)	*p*	HR (95% CI)	*p*	HR (95% CI)	*p*
Physical activity						
SB	1.00 (Reference)		1.00 (Reference)		1.00 (Reference)	
aPA	0.83 (0.59–1.17)	0.29	0.85 (0.61–1.19)	0.349	0.85 (0.61–1.20)	0.353
haPA	0.60 (0.41–0.86)	0.006	0.66 (0.45–0.97)	0.033	0.67 (0.46–0.99)	0.042

*Note*: Model 1, crude; Model 2, adjusted for age, sex, marital status, alcohol consumption, smoking status, and education level; Model3, adjusted for age, sex, marital status, alcohol consumption, smoking status, education level, and history of diabetes, and cancer.

Abbreviations: aPA, active physical activity; CI, confidence interval; haPA, highly active physical activity; HR, hazard Ratio; SB, sedentary behavior.

**FIGURE 5:**
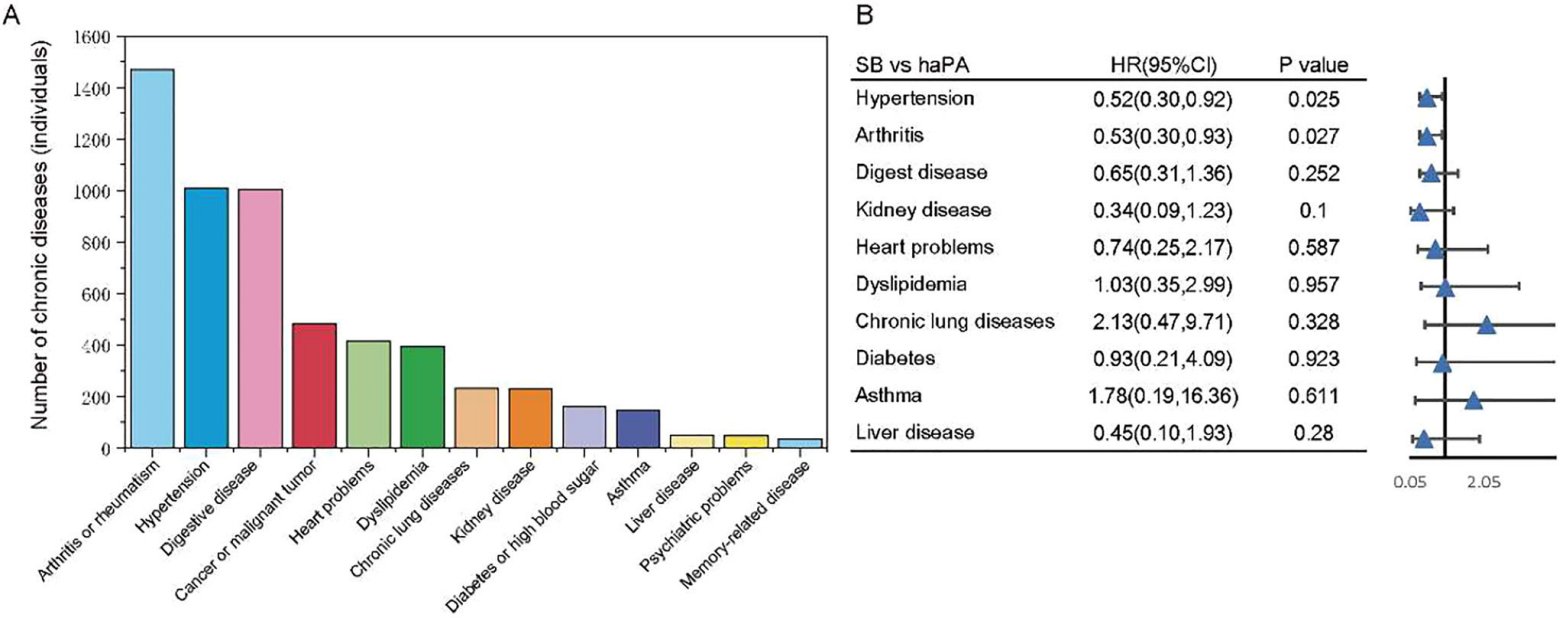
A) Participants with different histories of chronic diseases at baseline survey. (B) Association between different types of physical activity and risk of first stroke in people with a history of different illnesses. SB, sedentary behavior; haPA, highly active physical activity; HR, hazard Ratio; CI, confidence Interval.

The results of this study indicated a high prevalence of chronic diseases, such as hypertension and arthritis, among middle‐aged and elderly individuals in China in 2011. In addition, we further investigated the impact of exercise therapy on the risk of a first stroke in individuals with chronic conditions (Figure 5). In the subgroup analysis of patients with hypertension, those participating in haPA demonstrated a reduced HR for the incidence of first stroke, with an HR of 0.57 (95% CI, 0.33–0.99). Upon adjustment for confounding factors, the HR for the first stroke within the hypertension subgroup remained statistically significant at 0.52 (95% CI, 0.30–0.92) (Table S4 and Figure 3A). In addition, we conducted multi‐model cox regression analyses for eight other chronic conditions and found that aPA and haPA reduced the risk of first stroke in participants with chronic conditions of arthritis (Table S3 and Table S4).

**FIGURE 6:**
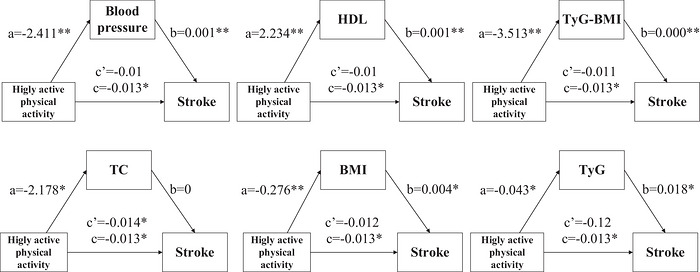
Mediation of the relationship between highly active physical activity and first stroke by different cardiovascular indicators. Path a: effect of highly active physical activity on each cardiovascular marker. Path b: effect of each cardiovascular marker on first stroke risk. Path c: total effect (before adjusting for mediators). Path c′: direct effect (after adjusting for mediators). BP, blood pressure; HDL, high‐density lipoprotein; TyG‐BMI, triglyceride‐glucose‐body mass index; TC, total cholesterol; BMI, body mass index; TyG, triglyceride‐glucose index. Adjustments were made for age, gender, marital status, educational background, place of residence, alcohol consumption history, smoking status, and history of diabetes.

Figure 6 illustrates the results of the mediation analysis. Following adjustment for all covariates, cardiovascular indicators were assessed as mediators to determine the direct or indirect impact of haPA on the initial occurrence of the first stroke. Overall, Table S5 indicates that the indirect effects of haPA on first stroke through blood pressure, HDL, BMI, TyG, and TyG‐BMI were statistically significant, suggesting that these cardiovascular markers may partially account for the observed association.

**FIGURE 7:**
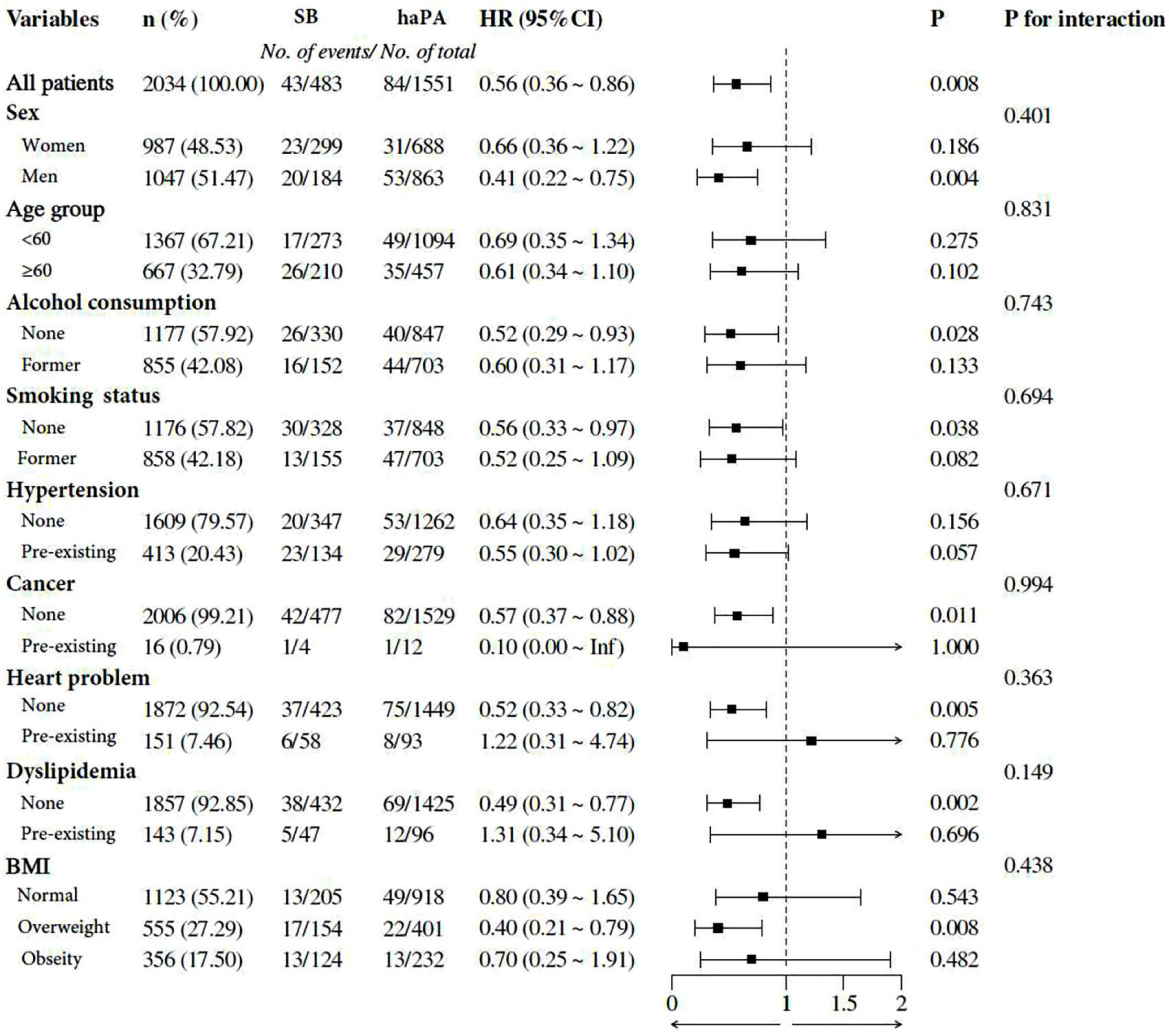
Relationship between first stroke and type of physical activity: Subgroup analysis by baseline characteristics. Hazard ratios (HRs) and 95% CIs for very active versus sedentary physical activity were estimated after adjusting for place of residence, marital status, diabetes, and lipids. After adjusting for sex, age, smoking, alcohol consumption, body mass index, total smoking, alcohol consumption, hypertension, cancer, heart disease, dyslipidemia, and body mass index were analyzed in subgroups. Interactions were compared by likelihood ratio tests comparing highly active physical activity with a model with a sedentary (continuous) multiplicative interaction term.

In the stratified analysis (Figure 7), highly active physical exercise among middle‐aged and older participants was associated with a reduced risk of first stroke. Compared to sedentary behavior, lower risks of first stroke were observed in males, nonsmokers, nondrinkers, individuals without cancer, those without heart disease or dyslipidemia, and among the overweight population. However, in the overall aPA group, no significant reduction in the risk of first stroke was observed compared to the sedentary group. Similarly, the stratified analysis did not demonstrate a decreased risk of first stroke in the aPA group relative to the sedentary group.

## 4 Discussion

To our knowledge, this is the first study to use nationally representative data to investigate the longitudinal association between physical activity and the first occurrence of stroke among middle‐aged and elderly Chinese community residents over 45 years of age. Both cohort and cross‐sectional studies have found a negative correlation between haPA and first stroke. We found that about 35% of participants who engaged in haPA were associated with a reduced risk of first stroke, demonstrating the positive effects of highly active physical exercises such as farming, aerobics, and fast cycling. A prospective study of 14,599 middle‐aged and elderly individuals from the general UK population found that those with high baseline physical activity had a 42% lower risk of stroke compared to those who were persistently inactive individuals, and benefits were gained from any physical activity (Mok et al. 2019). Furthermore, another meta‐analysis of 752,050 follow‐up participants also found that all levels of leisure‐time physical activity were beneficial in preventing stroke (De Santis et al. 2024). Notably, our study did not find that all kinds of physical activity could prevent stroke, possibly due to the exclusion of occupational physical activities.

In the national stroke prevalence distribution map over the 4 years of this study, it was observed that the prevalence of stroke increased annually, with a more pronounced rise in the northern regions compared to the southern regions. The two cities with the greatest increase in stroke prevalence were located in the north, which may be attributed to differences in physical activity levels, influenced by climatic and dietary factors unique to the north (Lv et al. 2017). The two provinces with the smallest increase were Beijing and Shanghai, both of which are characterized by higher socioeconomic status, better education, and a more balanced diet and physical activity regimen (Ru et al. 2019).

Aerobic exercise may help prevent increases in blood pressure by improving insulin sensitivity and regulating autonomic nervous system function (Moraes‐Silva et al. 2013). Several studies have demonstrated that an elevated TyG index is associated with an increased risk of stroke (Wu et al. 2024). The biological mechanisms by which haPA can protect the cardiovascular system may be attributed to the fact that aerobic exercise can induce anti‐atherosclerotic adaptations in vascular function and structure through hemodynamic stimuli (Fiuza‐Luces et al. 2018). Physical activity can increase vascular endothelial shear stress, which stimulates vasodilation by releasing nitric oxide (NO) (Erkens et al. 2017). Regular physical activity can also reduce chronic inflammation by releasing myokines from skeletal muscles (Fiuza‐Luces et al. 2013). In our study, haPA was found to reduce the risk of first stroke among the hypertensive population, but was not significant in other chronic disease populations. A meta‐analysis including 94 randomized controlled trials found that behavioral counseling interventions for adults with cardiovascular risk factors, such as hypertension and dyslipidemia, were associated with a 20% reduction in cardiovascular risk (O'Connor et al. 2020). Another behavioral counseling intervention found that the net benefits for CVD risk reduction were minimal among adults without CVD risk factors (US Preventive Services Task Force et al. 2022). Therefore, participants with cardiovascular risks are more likely to benefit from physical exercise.

The current study indicates that the protective effects of exercise on cerebrovascular diseases are linked to the aforementioned factors, as further mediation analysis demonstrates that high‐intensity physical activity in the elderly may reduce the risk of a first stroke by regulating hypertension and dyslipidemia, including HDL‐c. Research by (Mora et al.^2007^) in a prospective study of 27,055 apparently healthy women found that the inverse relationship between physical exercise and CVD risk was largely mediated by known risk factors, primarily due to inflammatory/hemostatic biomarkers and blood pressure (Mora et al. 2007).

Our study demonstrates that, in both the overall population and within specific gender groups, the risk of a first stroke decreases progressively with increasing total physical activity per week. A linear dose–response relationship was observed between aPA and the risk of a first stroke, with the risk decreasing as the total amount of physical activity increased within the aPA group. In the high‐intensity physical activity (haPA) group, physical activity exerted a protective effect against first stroke up to a threshold of 17,000 MET‐min per week. Beyond approximately 17,000 MET‐min/week, the point estimate trended upward; however, CIs were wide in this upper range (Hou et al. 2021; Thompson et al. 2007; Cameron et al. 2010). Our study shows that haPA has a more pronounced effect on reducing stroke risk in men, possibly due to the loss of ovarian hormones during menopause in women and the additive effect of atrial fibrillation on their stroke risk (Branyan and Sohrabji 2020; Peters et al. 2019). However, a cohort study following 9.2 million Canadian adults over a median of 15 years assessing the relationship between age and gender and stroke incidence found that women aged 40–80 had a lower incidence of stroke (Vyas et al. 2021). In addition, participants without heart disease, those who were overweight, and those without dyslipidemia were more likely to benefit from highly active exercise.

Our analysis also discussed the impact of total physical activity volume on the risk of the first stroke. Interestingly, at low levels of total physical activity, the risk of stroke quickly decreased with increasing total physical activity. This is consistent with previous meta‐analyses that increased physical activity volume is associated with reduced stroke risk (Kyu et al. 2016). Furthermore, the World Health Organization's Physical Activity and Sedentary Behavior Guidelines Task Force recommended optimal health‐related physical activity levels for children and adolescents, adults, and older people to address global health disparities (DiPietro et al. 2020).

### 4.1 Limitations

Several factors may limit the generalizability of these findings. First, physical activity was measured only at baseline (Wave 1, 2011) without repeated assessments during follow‐up. Second, a substantial proportion of total physical activity in CHARLS derives from occupational activities such as farming, which may differ physiologically from the leisure‐time exercise predominant in Western cohorts (Holtermann et al. 2012). Third, dietary sodium intake differs markedly between northern and southern China, and this regional dietary pattern may modify the physical activity and stroke relationship (Huang et al. 2020). Fourth, CHARLS does not distinguish between ischemic and hemorrhagic stroke subtypes, which are distributed differently across populations and may respond differentially to physical activity (M.‐H.Wang et al. 2024; Chen et al. 2025).

## 5 Conclusions

In this prospective cohort of middle‐aged and older adults, haPA was associated with a lower risk of first stroke. Mediation analyses suggested that cardiovascular biomarkers partially accounted for this inverse association.

## Author Contributions

**Guanzhao Wu**: funding acquisition, investigation, supervision, data curation, writing – review and editing, formal analysis. **Zetai Bai**: validation, software, formal analysis, data curation, conceptualization, project administration, methodology, writing – review and editing, writing – original draft. **Huiping Bai**: conceptualization, methodology, software, project administration, writing – original draft.

## Funding

This study was supported by the Young Taishan Scholars tsqn (No. 202306352) and the Qilu Young Scholars Program of Shandong University (SDQLQN2021‐01). The funding sources had no role in the design and conduct of the study; collection, management, analysis, and interpretation of the data; preparation, review, or approval of the manuscript; and decision to submit the manuscript for publication.

## Conflicts of Interest

The authors declare no conflicts of interest.

## Supporting information




**Supplementary Materials**: brb371601‐sup‐0001‐SuppMat.docx

## Data Availability

Data and codes used in this study are available on reasonable request to the authors.
