# Supplementary material for: Impact of Physical Activity on Stroke Risk in Middle‐Aged and Older Adults
Source: Brain Behav. 2026 Jul 14;16(7):e71601. doi: 10.1002/brb3.71601 (PMC13366402; doi:10.1002/brb3.71601)
Supplement: Supplementary file 1 — Supplementary Materials: brb371601‐sup‐0001‐SuppMat.docx [file BRB3-16-e71601-s001.docx]

**Impact of Physical Activity Intensity on Stroke Risk in Middle-aged and Older Adults**

Huiping Bai ^a^, Zetai Bai ^c,*^, Guanzhao Wu ^b,c,*^

*^a^ Qilu Hospital (Qingdao), Cheeloo College of Medicine, Shandong University, 758 Hefei Road, Qingdao, China*

*^b^* *Department of Central Laboratory, Qilu Hospital (Qingdao), Cheeloo College of Medicine, Shandong University, Qingdao, Shandong, 266035, China*

*^c^ Qilu Hospital, Cheeloo College of Medicine, Shandong University, Jinan 250012, Shandong, China*

**^*^Correspondence to:** Dr. Guanzhao Wu, Email: [guanzhao.wu@email.sdu.edu.cn](mailto:guanzhao.wu@email.sdu.edu.cn), ORCID: 0000-0002-8170-7993

**Figure S1. Relationship between first stroke and active physical activity (aPA): Subgroup Analysis by Baseline Characteristics**


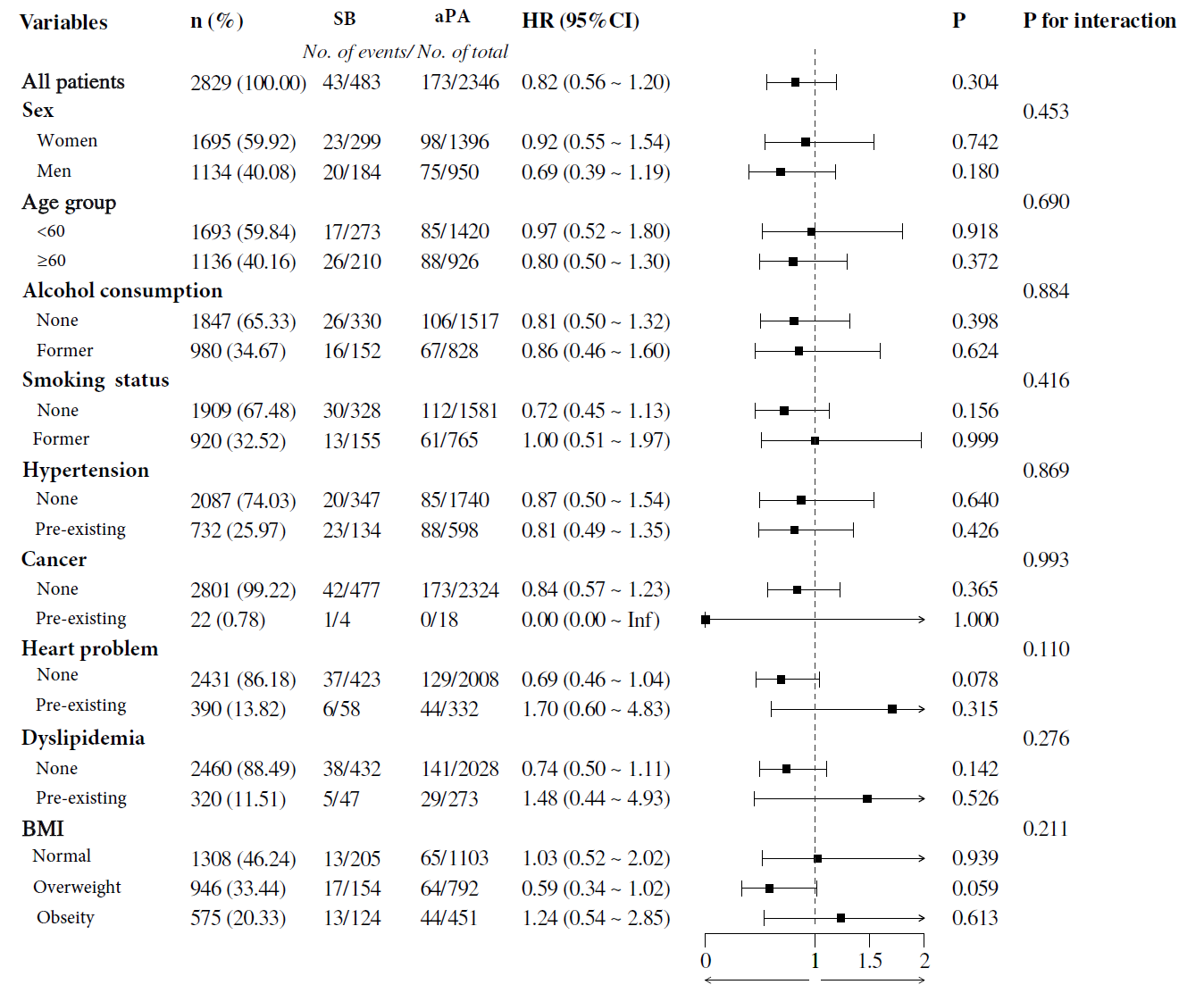


Hazard ratios (HRs) and 95% CIs for very active versus sedentary physical activity were estimated after adjusting for place of residence, marital status, diabetes, and lipids.

after adjusting for sex, age, smoking, alcohol consumption, body mass index, total

smoking, alcohol consumption, hypertension, cancer, heart disease, dyslipidemia and body mass index were analyzed in subgroups. Interactions were compared by likelihood ratio tests comparing active physical activity with a model with a sedentary (continuous) multiplicative interaction term.

**Table S1. Factors Influencing Stroke Risk in 2015: Univariate and Multivariate Regression Analyses**

Abbreviations: SB,Sedentary behavior; aPA, active physical activity; haPA, highly active physical activity; OR, Odds Ratio; CI, Confidence Interval;

**Table S2. Factors Influencing Stroke Risk in 2018: Univariate and Multivariate Regression Analyses**

Abbreviations: SB, Sedentary behavior; aPA, active physical activity; haPA, highly active physical activity; OR, Odds Ratio; CI, Confidence Interval.

**Table S3.** **Association of physical activity intensity with first stroke in participants with chronic disease: multi-model cox risk regression**

Abbreviations: SB, Sedentary behavior; aPA, active physical activity; haPA, highly active physical activity; HR, Hazard Ratio; CI, Confidence Interval.

Model1: crude.

Model2: adjusted for age, sex.

Model3: adjusted for age, sex, marry status, alcohol consumption, smoking status,education level.

**Table S4. Association between different types of physical activity and risk of first stroke in people with a history of different illnesses.**


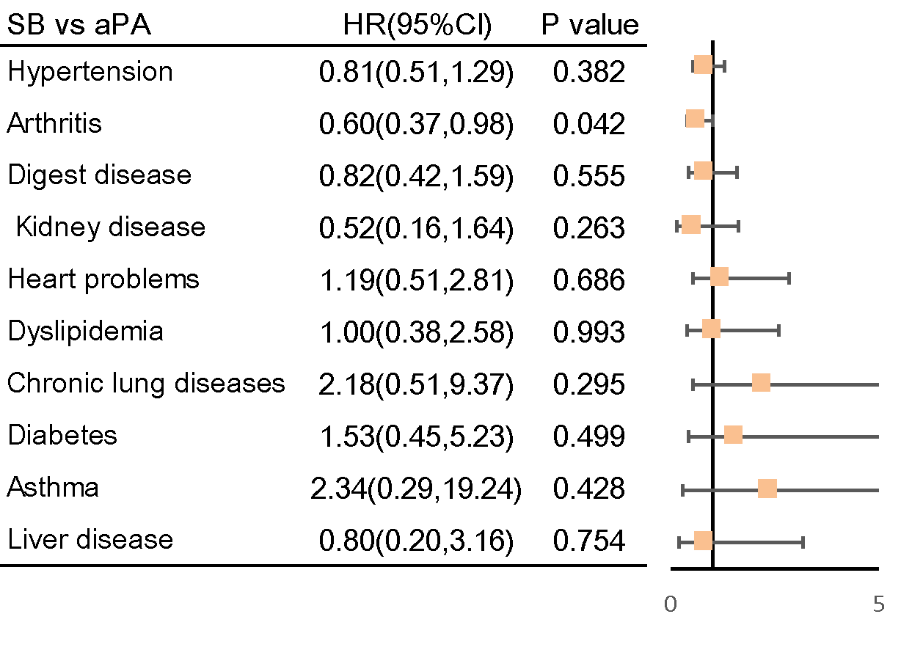


Abbreviations: SB, Sedentary behavior; haPA, highly active physical activity; HR, Hazard Ratio; CI, Confidence Interval.

**Table S5. Mediation Analysis of the Association Between Highly active Physical Activity and Risk of First Stroke via Cardiovascular Markers**

Abbreviations: BP, Blood Pressure; HDL, High-Density Lipoprotein; TyG-BMI, Triglyceride-Glucose-Body Mass Index; TC, Total Cholesterol; BMI, Body Mass Index; TyG, Triglyceride-Glucose Index. Adjustments were made for age, gender, marital status, educational background, place of residence, alcohol consumption history, smoking status, and history of diabetes.
